# Supplementary material for: Synchronization of gene expression across eukaryotic communities through chemical rhythms
Source: Nat Commun. 2021 Jun 29;12:4017. doi: 10.1038/s41467-021-24325-z (PMC8242030; doi:10.1038/s41467-021-24325-z)
Supplement: Supplementary file 1 — Supplementary Information [file 41467_2021_24325_MOESM1_ESM.pdf]

Supplementary Information for

Synchronization of gene expression across eukaryotic communities through chemical rhythms

Sara Pérez-García[1,2], Mario García-Navarrete[1,2], Diego Ruiz-Sanchis[1], Cristina Prieto-Navarro[1], Merisa Avdovic[1], Ornella Pucciariello[1], Krzysztof Wabnik[1,*]

[1]Centro de Biotecnologıa y Genomica de Plantas (Universidad Politecnica de Madrid – Instituto Nacional de Investigacion y Tecnologıa Agraria y Alimentaria), Autopista M-40, Km 38 – 28223 Pozuelo de Alarcon, Spain

*Correspondence should be addressed to: k.wabnik@upm.es

[2]These authors contributed equally: Sara Pérez-García and Mario García-Navarrete.

Supplementary Information contains:

Supplementary Methods

Supplementary Figures 1-15

Supplementary Table 1

Supplementary Methods

Microfluidic Mold fabrication. Molds for the production of microfluidic devices were designed in InkScape and printed on plastic sheets with the monochrome laser printer at 1200dpi resolution. A density of Ink deposition was used to control the feature height. Plastic wafers were cut and transferred to the thermal oven set to 160°C to shrink by one-third of the original size, the baked again for 10 min to smoothen and harden the ink. Finally, molds were cleaned with soap, rinsed with isopropanol and DDI water and dry using a nitrogen gun and secured with Scotch tape before use.

Soft Lithography. Molds were introduced in plastic 90mm Petri dishes and fixed with double-sided tape. Dowsil Sylgard 184 Polydimethylsiloxane (PDMS) was properly mixed in a 10:1 (w/w) ratio of elastomer and curing agent and stirred until the uniform consistency was achieved. Approximately 27mL of the homogeneous mixture was poured in each petri dish and completely degassed using the 8 CFM 2-stage vacuum pump for approximately 20 minutes. Degassed PDMS was cured at 80°C for 2h. Cured PDMS was removed from the petri dish, separated from the wafer and cut to extract the individual chips. Fluid access ports were punched with 0.7mm diameter World Precision Instruments (WPI) biopsy puncher and flushed with ethanol to remove any remaining PDMS. Individual chips were cleaned with ethanol and DDI water and Scotch tape to remove any remaining dirt particles.

Microfluidic device bonding. At least one day before use, individual chips and coverslips were cleaned in the sonic bath and rinsed in ethanol, isopropanol, and water. Both surfaces were exposed to Corona SB plasma treater (ElectroTechnics Model BD-20AC Hand-Held Laboratory Corona Treater) between 45 seconds to 1 minute, then surfaces were brought together and introduce at 80°C in an oven overnight to obtain the enhanced bond strength.

Calibration of microfluidic mixer module. Mixer Calibration was performed by staining one of two inputs with Rhodamine B 0.001% (w/v). A pair of 60mL syringes were prepared with 20mL of water or water + dye. Three 50mL falcon tubes were filled with 20mL of water and connected to waste and cell loading ports. Syringes and falcon tubes were set on linear actuators which control pressure in the microfluidic device through customized software. The microfluidic device was vacuumed for at least 20 minutes to remove air bubbles and facilitate device wetting before connecting Tygon microbore tubing 0.020" x 0.060"OD (ColePalmer Inc.). After air removal, one syringe was connected and then the other syringes were plugged sequentially once liquid reached and filled the ports. Switching between inputs was regulated with the gravity-aided hydrostatic pressure by changing the relative height of syringes. Increasing

height of input media 1 (M1) over input media 2 (M2) produces the pressure difference in the mixing module. Therefore, at maximum height differences, microfluidic chip is filled with 100% of M1 and 0% of M2. Syringe positions were adjusted with 0.1 mm precision by changing height to obtain highly efficient mixing between 0% and 100% . The excessive flow is diverted towards an auxiliary waste output (W1) that releases the pressure from the mixer. Flow then was introduced into the second chaotic mixer to assure a rapid switching of conditions. The calibration process was repeated at least three times with three independent microfluidic devices. Maximum and minimum height settings varied solely by a few millimeters, therefore we used averaged measures without cross-contaminating media inputs, which were following: 610 mm for the maximum and 390 mm for minimum settings, respectively. All three replicates showed exact 50% mixing for the 500 mm settings.

Cell loading procedure. All tubing lines were sterilized with ethanol and plugged to syringes or introduced in falcon tubes under sterile conditions. Fresh yeast colony was grown in low fluorescence media composition (Formedium, UK) with 2% sucrose as a carbon source or 2% glucose (K1 toxin producing strain). The next day, yeast cultures were diluted 10-20 times approximately to 0.2-0.4 $O.D_{600}$ to obtain highly concentrated cells that were transferred to a 50mL falcon tube for loading. 60mL media syringes were filled with 25mL of inducing media (2% sucrose + 0.5%galactose) with or without compounds and 50mL waste falcons tubes were filled with 10mL of DDI water. Before loading, devices were vacuumed for at least 20 minutes to remove all the air from the channels and traps. Syringes and falcon tubes were placed on the height control system and lines were connected as follows; media syringes were plugged first and kept above all other inputs to prevent media contamination. Adjusting the height of the cells-containing falcon tube as well as media and waste aids in controlling the cell seeding in the traps. Although many cells pass through the chip directly towards the waste port, few cells got captured via micro valves and seeded the traps. Once 10-20 cells were captured in each trapping region, the flow from the cell loading port was reverted by decreasing the height to the same level as for the auxiliary waste.

Mathematical modeling. To derive a simple mathematical model of mean switching frequency (MSF) we used formulations[44,45] with following modifications. Briefly, we hypothesize that hysteresis region (ΔH) increases with input frequencies (ω_0) (rate-dependent hysteresis):

$$\Delta H(\omega_o) = \Delta H_o + e^{-\frac{\beta}{\omega_o}} \tag{1}$$

where ΔH_o=0.1 is initial size of hysteresis region and β=0.01 is an expansion factor.

According to the theory of stochastic switching in noisy bistable systems[44, 45], MSF can be approximated by a following formula:

$$MSF = \frac{\pi}{2} \cdot \left[r_1 + r_2 + 2 \cdot \omega_o - \sqrt{(r_1 - r_2)^2 + 4 \cdot \omega_o^2} \right] \quad \textbf{(2)}$$

where, r_1 and r_2 are rates of the switch defined as[44, 45]:

$$r_1 = r_0 \cdot e^{-\frac{\Delta H(\omega_O)+A}{\xi}}, r_2 = r_0 \cdot e^{-\frac{\Delta H(\omega_O)-A}{\xi}} \quad \textbf{(3)}$$

Parameters r_0=1 is basal switching rate and A is driving amplitude.

We altered driving amplitude (A) and frequency (ω_0) to study the robustness of synchronization for the increasing noise strength (ξ) as shown in Supplementary Fig. 12.

Supplementary Figures

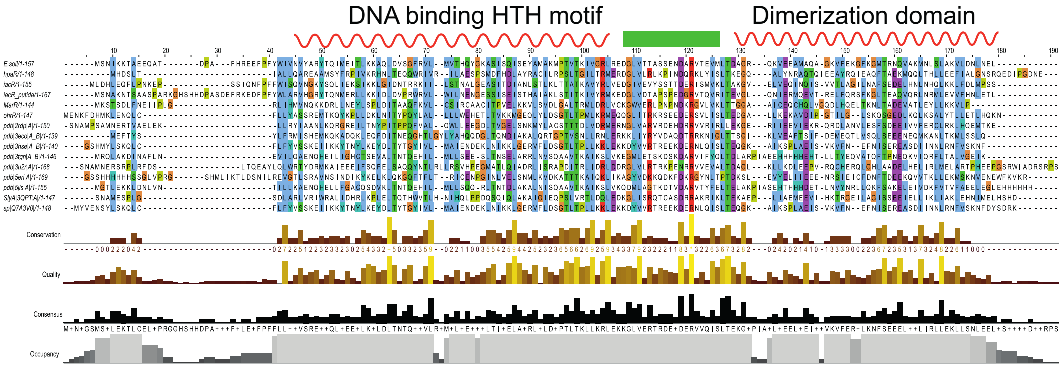

Supplementary Fig. 1. Structure-based alignment of members from the family of Mar proteins. The structural alignment of MarR-type transcriptional repressors highlights structurally conserved regions such as DNA binding and dimerization interfaces α-helix (red) : β-sheet (green) : α-helix (red).

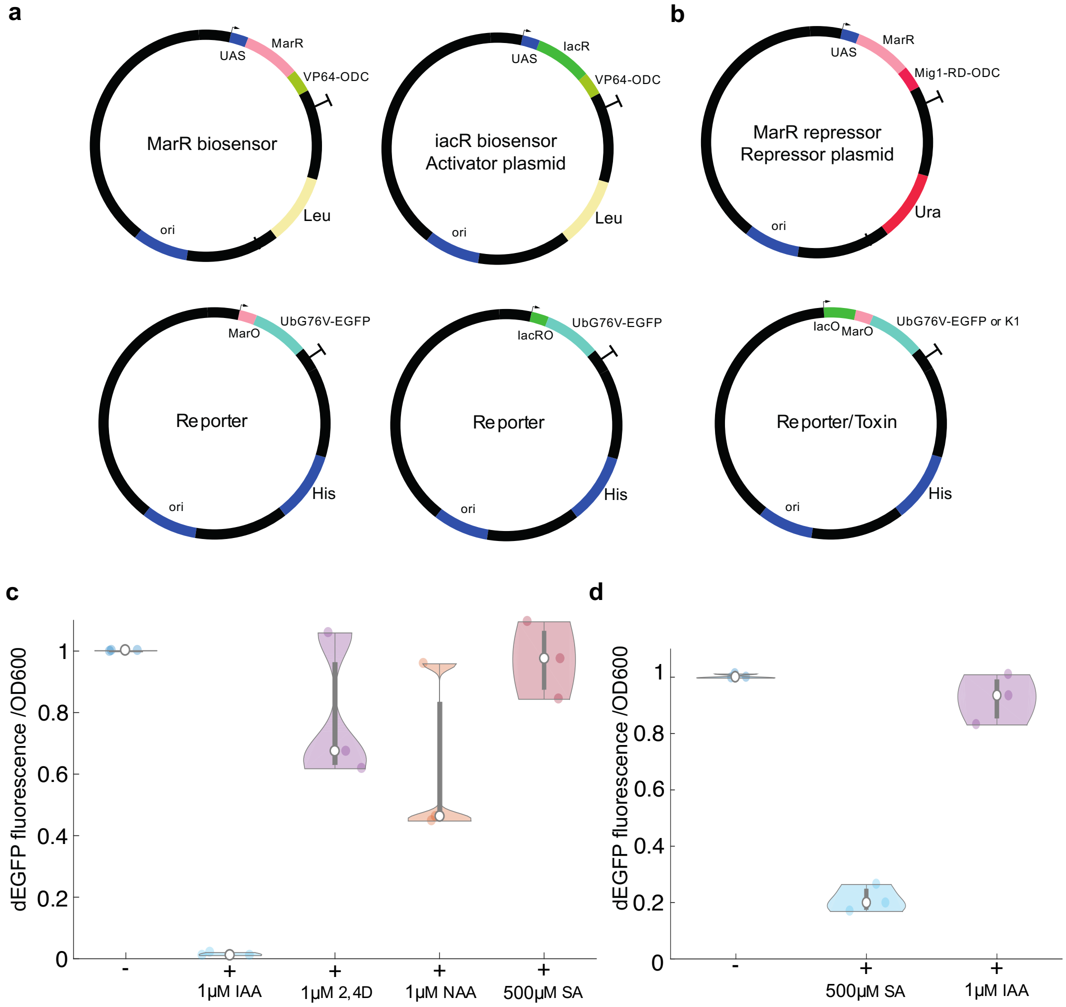

Supplementary Fig. 2. System constructions and specificity of Mar receptors to IAA and SA. (**a**), Plasmid compositions include all parts used to construct synthetic gene circuits and fluorescent dEGFP reporter (UbG76V-EGFP) or K1 toxin (K1). UAS denotes GAL4 binding sites in Galactose inducible promoter. *MarO* and *IacO* are operator sequences for MarR and IacR transcriptional regulators. (**b**), The construction of MarR repressor and reporter plasmids are shown. MarR was tagged with the last 24 aa of MIG1 transcription factor that binds the general yeast co-repressor complex[48]. (**c**), Orthogonality and high specificity of IacR module to IAA but not SA or other synthetic auxins (2,4D and NAA). (**d**), MarR module is insensitive to IAA. Data from three independent replicates per condition (n=3) are shown. The description of violin plots is as in Fig.1f.

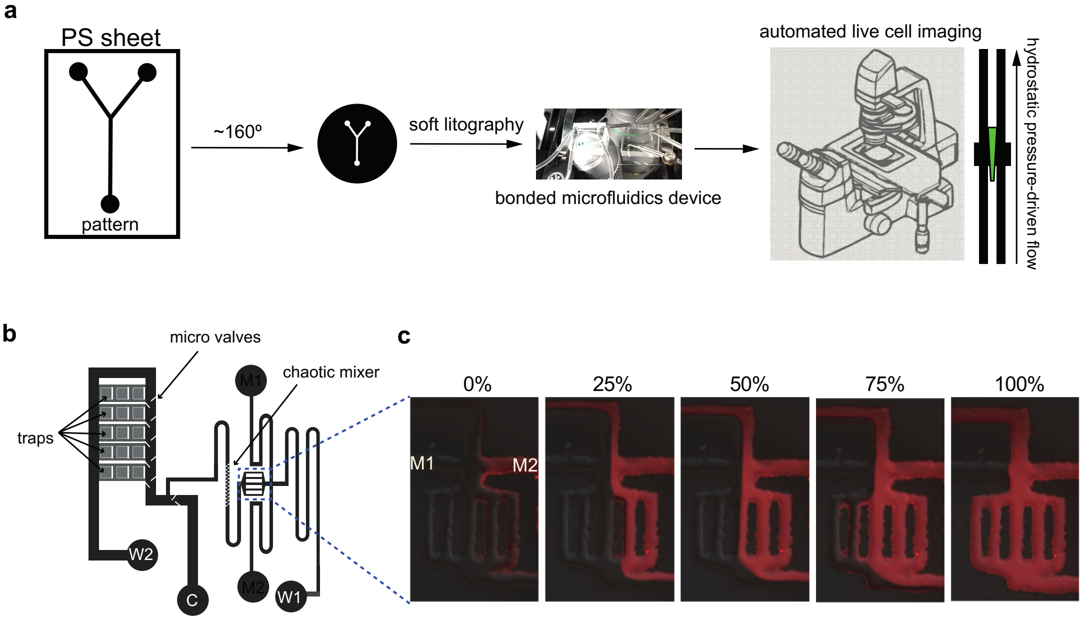

Supplementary Fig. 3. Microfluidics design and flow control scheme. (**a**), A simplified process of microfluidic device fabrication. Initially, the pattern of channels is printed with a laser printer at 1200dpi resolution on plastic polystyrene (PS) sheet. Next, a PS sheet is baked at approx. 160^0C such that it shrinks around 70% in x-y and increases in height by 10-fold. The height of channels and traps can be controlled by the density of ink deposition to create cell monolayers. Soft lithography is used to make PDMS silicon devices that are then bonded to the cover glass to finalize the device. Microfluidics platform integrates automated epifluorescence microscope and customized microfluidic flow system with linear actuators which allows the gravity-regulated hydrostatic pressure control. (**b**), A final design of microfluidic chip for experiments. Channels widths were 120μm for in the mixer module and 500μm in main channels. The approximate height of channels was 25 microns. Cell traps had 500μm x 500μm size and height of approximately 7 microns. M1 and M2 are media ports that supply media with or without SA or IAA. W1 is a media waste port that controls an influx in the mixer module and W2 is a general waste port. Cells are loaded through C port and seeded in the trapping region with the help of microvalves. (**c**), Screenshots from mixing conditions and 3-step mixer calibration (0%, 50%, 100%) with Rhodamine B red fluorescent dye.

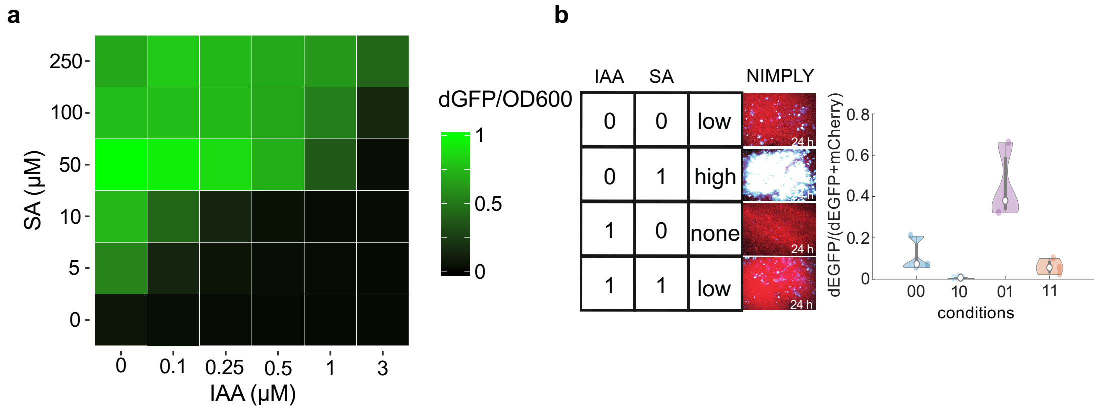

Supplementary Fig. 4. Response landscape of combined circuit with MarR and IacR receptors. (**a**), SA and IAA concentration scans reveled transitions between four boundary states (0-0; 1-0;0-1; 1-1) (average of triplicates is shown). Color coding map for normalized dEGFP fluorescence is shown. (**b**), NIMPLY logic gating response of circuit presented in Fig 1G. in microfluidics experiments. Examples of screenshots of fluorescence markers after 24 hours of growth in microfluidics traps per condition are shown. Three replicates (n=3) per condition are presented. The description of violin plots is as in Fig.1f.

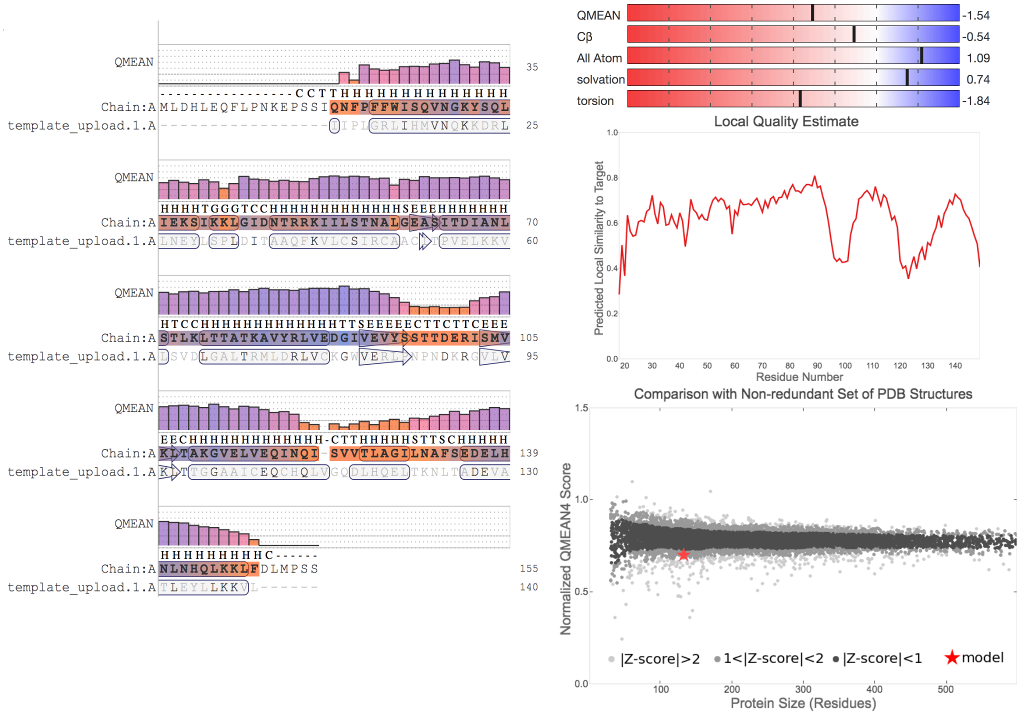

Supplementary Fig. 5. Quality assessment of predicted IacR structure. Left panel, the structural alignment with *E.coli* MarR templates showed a high quality structure prediction (QMEAN score, SWISS-MODEL, https://swissmodel.expasy.org/qmean/) in particular, for α-helix regions that cover DNA and ligand binding domains. Right panel, IacR prediction assessment based on several local estimators. Note similarity of IacR to MarR is near 60%. Z-score (the energy separation between the native fold and the average of an ensemble of misfolds in the units of the standard deviation of the ensemble metric against all crystalized structures); IacR model clusters between 1< Z-score < 2 which indicate the high confidence of predicted structure.

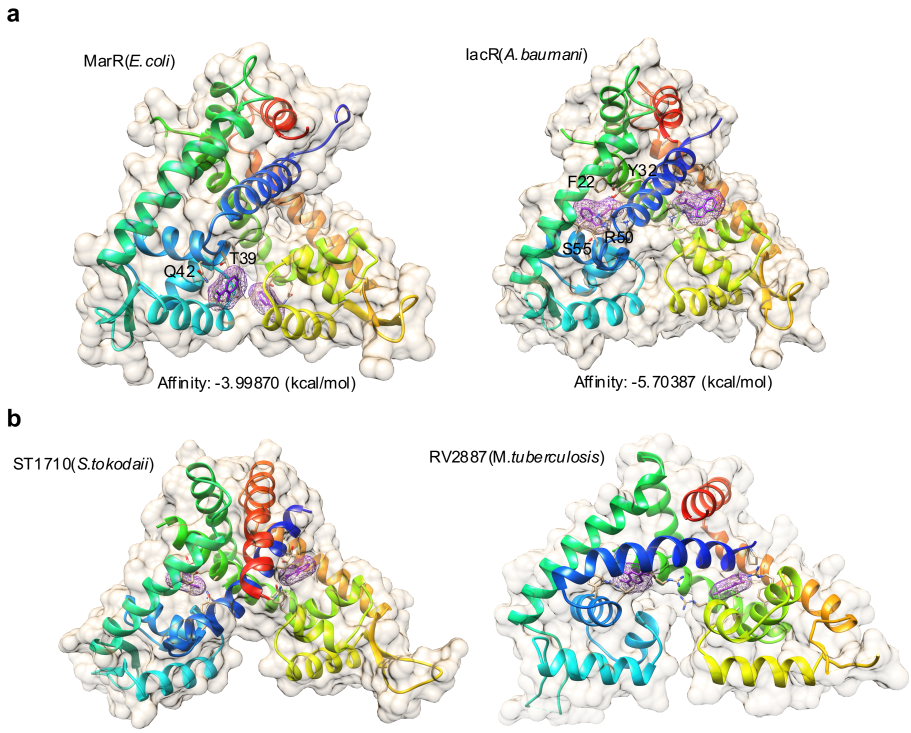

Supplementary Fig. 6. Comparison of IacR-IAA binding pose with other MarR-type receptors. (**a**), Binding pocket of MarR with SA (RCSB code: 1JGS [http://doi.org/10.2210/pdb1JGS/pdb])(left panel) and predicted IAA binding pocket of IacR(right panel) with key residues involved in hydrophobic and hydrogen ligand interactions. Gibbs free energy of binding (affinity) were calculated with AUTODOCK scoring function. (**b**), left panel, SA binding poses of ST1710 (RCSB code: 3GF2 [http://doi.org/10.2210/pdb3GF2/pdb]), and RV2887 (RCSB code: 5X80 [http://doi.org/10.2210/pdb5X80/pdb]) (right panel). Note the significant similarities between IacR and other MarR-type receptor including contacts with the first two α-helices.

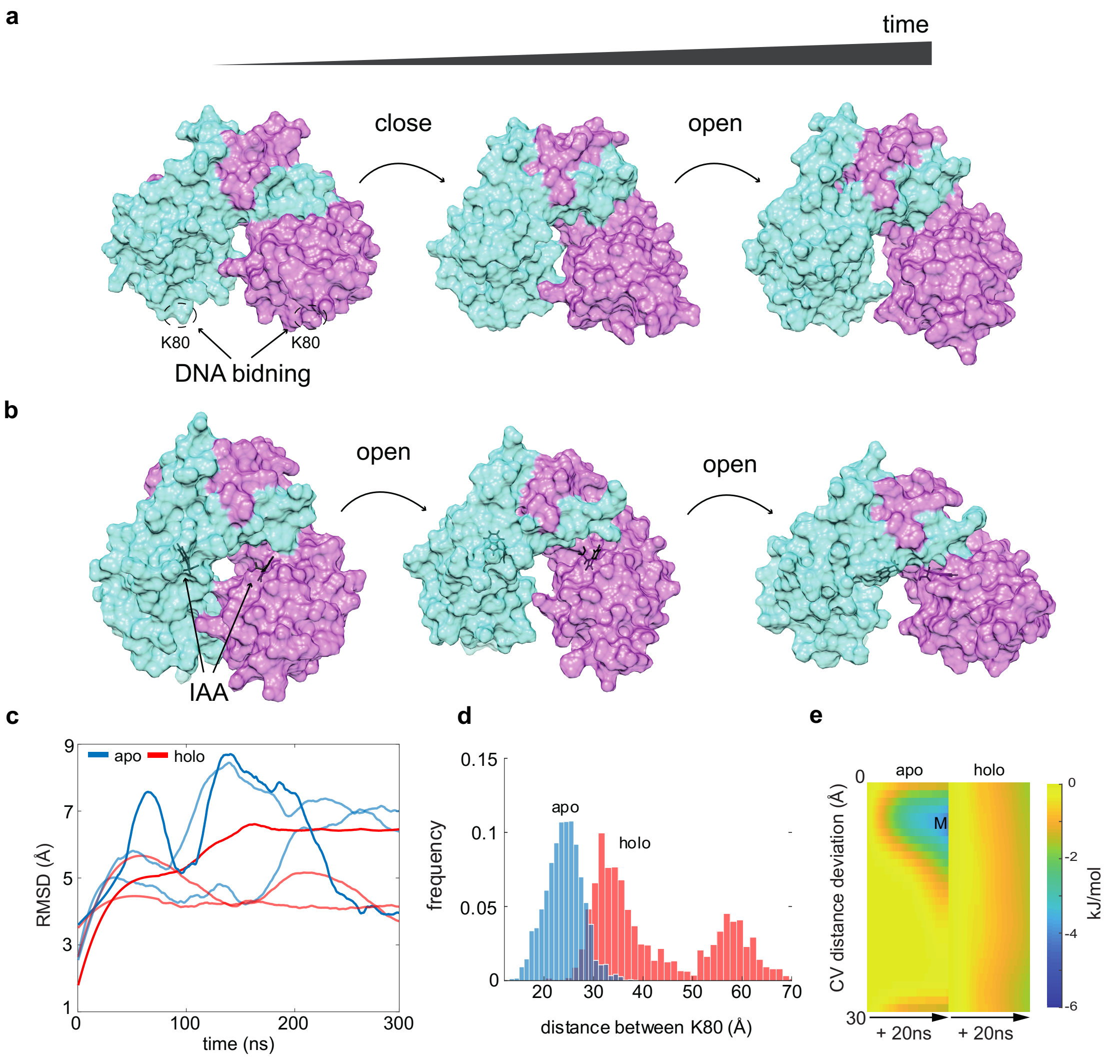

Supplementary Fig. 7. Predicted dynamics of IacR receptor. (**a, b**), Time-lapse from MD simulations of the predicted IacR apoprotein (a) (monomers shown in cyan and purple) and IAA-bound holoprotein (b). DNA binding charged K80 residues (equivalent to R73 in MarR) are shown. Note cycles of closure and opening in the apo configuration similar to those observed in MarR simulations (Fig 2a). Predicted dynamic poses of two IAA molecules along the time-course of MD simulation (b). Note, a progressive opening of the dimer (unlike $MarR_{holo}$ which closes) which pushes pair of DNA binding domain far enough to preclude potential DNA binding. (**c**), RMSD traces of IacR for apo (blue) and halo(red) configuration are shown; 3 independent replicates per modelled system are shown. The root-mean-square deviation of atomic positions (RMSD) is shown for dimer simulations of 300 ns. (**d**), A probability distribution of the mean distance between K80 atoms of both monomers for apo (blue) and holo(red) IacR configurations. Note that holo

state provides substantially longer distance between DNA binding domains compared to the apo state. (**e**), Energy landscapes for iacR configurations (apo, holo) for deviation from a putative distance of 20 A˚ between K80 (both chains) that could promote DNA binding. Heatmap of energy for apo and holo configurations indicate that unlike apoprotein, the IAA-bound holoprotein cannot reach the minimum required for association with the target DNA.

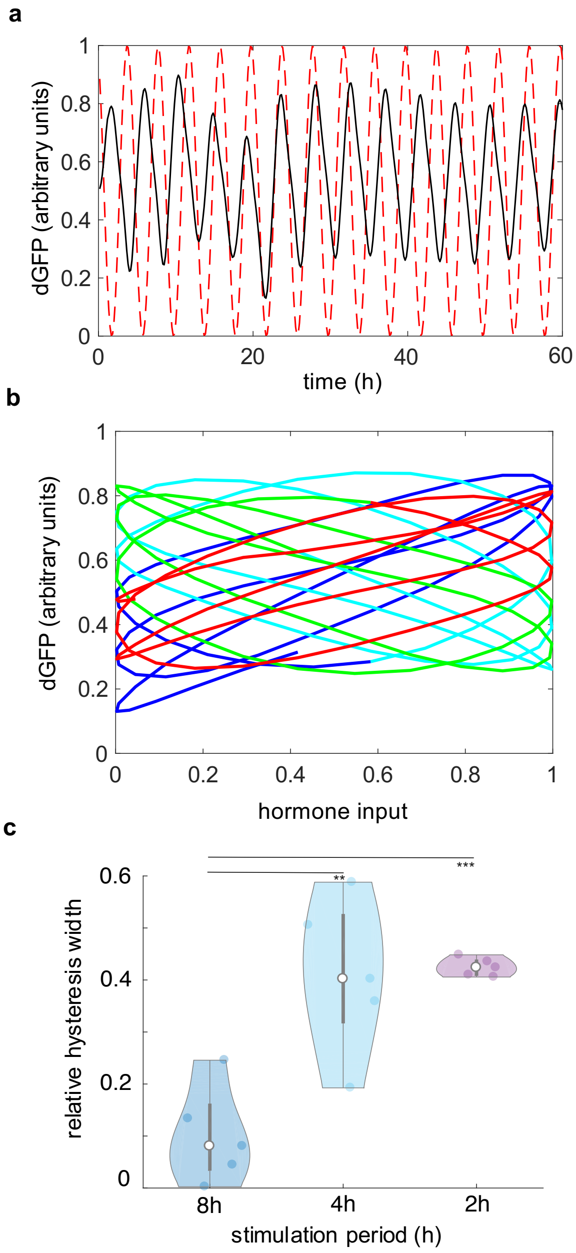

Supplementary Fig. 8. Dynamics of hysteretic switching observed in experiments. (**a**) Experimentally observed time trace of dEGFP fluorescence averaged over all communities (black line, same as Fig. 3c) and input stimulus (4h, 0-100%) shown as sinusoidal wave perceived by cells (red dashed line). (**b**) The same dEGFP reporter fluorescence (a) plotted against input stimulus (SA between 0% and 100%), Note characteristic lag between input and output (hysteresis loop) that switches between successive SA and IAA applications. The time evolution of switching events is color coded from blue (0h) to red (60h) and

corresponds to (a). (**c**) Relative increase in the width of hysteretic region was measured from the five successive peaks (n=5, violin plot) in the averaged time trace profiles (all communities) (a) for different frequencies of stimulus. (** p-value = 5.0^{-3}, *** p-value = 6.5^{-5}, a one-way ANOVA with post-hoc Tukey's HSD). The description of violin plots is as in Fig. 1f.

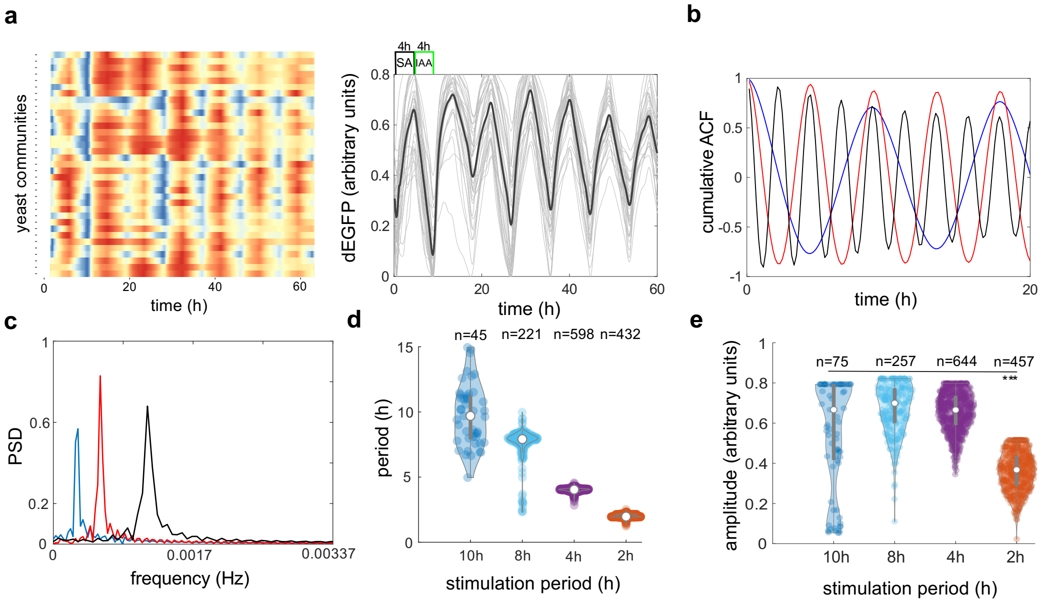

Supplementary Fig. 9. Robustness of synchronization based on frequencies of SA and IAA inputs. (**a**) Heatmap (left panel) and time profile of dEGFP (right panel) for 8-hour cycles between inputs. Note good level of synchronization between independent yeast communities. (**b**), Cumulative autocorrelation function (averaged over all recorded communities) for 8-hour (blue line), 4-hour (red line) and 2-hour (black line) of input frequencies. Note a remarkable synchronization potential of dual-receptor circuit. (**c**), Power spectra analysis reveals dominant frequencies of response that correspond to input frequencies. Color coding same as for (b). (**d**, **e**) While period of response gets sharper (d), amplitude eventually drops by 50% for 2-hour stimulus (e) following increased stimulus frequency (*** p-value = 1.1^{-10}, a one-way ANOVA with post-hoc Tukey's HSD). The description of violin plots is as in Fig. 1f. A heat map legend is as in Fig. 1i.

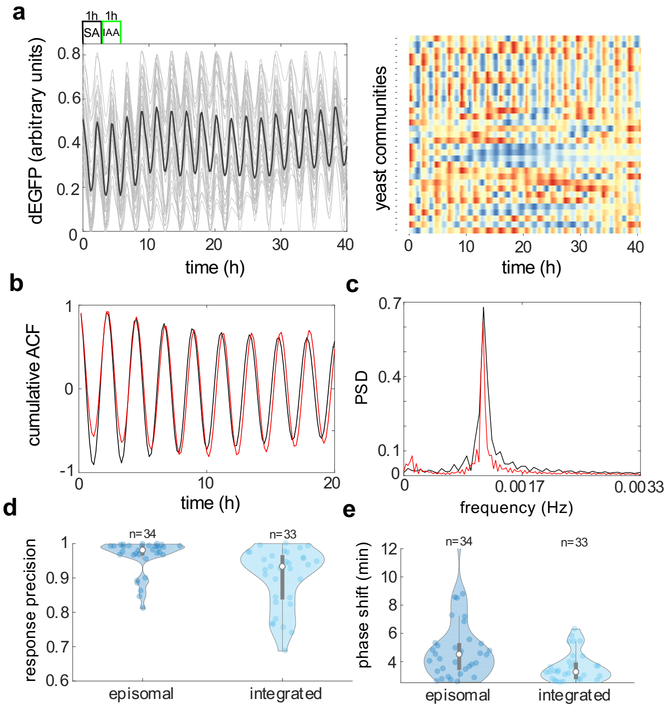

Supplementary Fig. 10. Robustness of synchronization mechanism to the Mar receptor copy number. **(a)**, Time evolutions of reporter fluorescence in spatially-distant yeast communities in the strain with the genomic integration of Mar-based circuit (single copy). Mean dEGFP trace for all communities is shown in black (left panel), and corresponding heat maps (right panel) for all recorded communities (n = 34, n = 33). (**b, c**), Cumulative autocorrelation function (b) and power spectra analysis (c) calculated from multicopy (black) and single-copy (red) circuit versions show the robust synchronous pattern at the inter-colony level regardless of copy number. (**d, e**), Response precision (d) and phase drift (e) as a function of copy number. The description of violin plots is as in Fig.1f. A heat map legend is as in Fig. 1i.

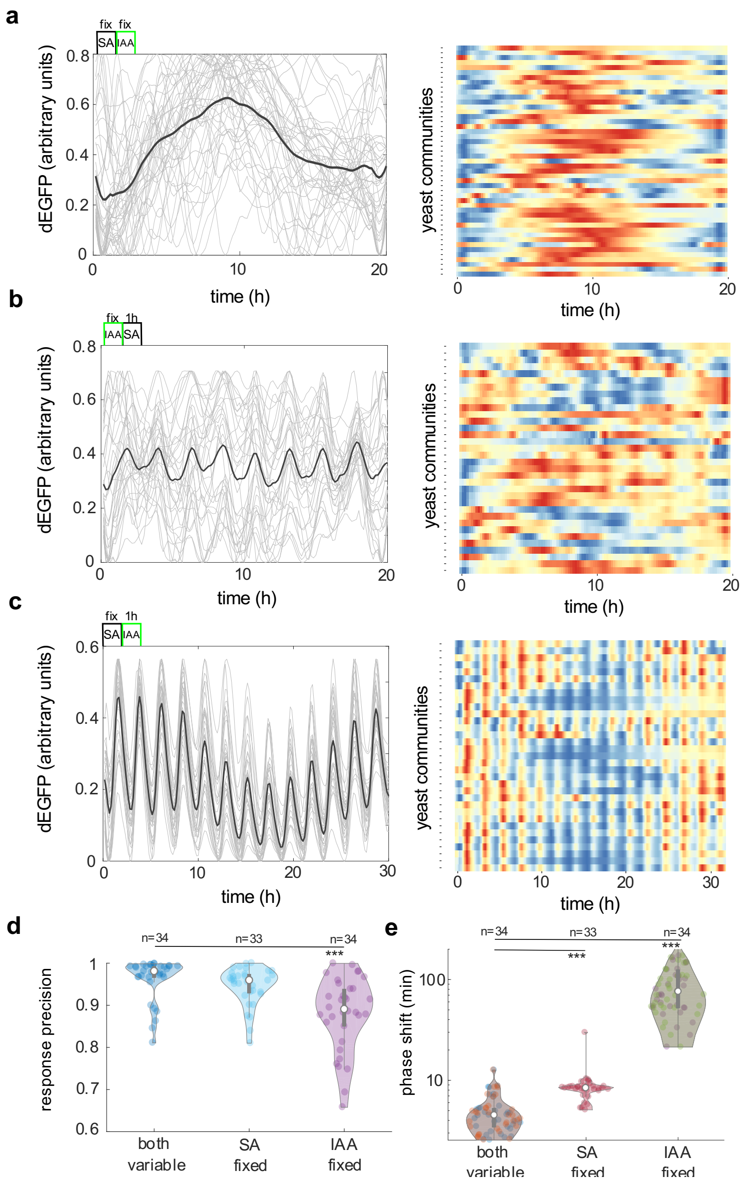

Supplementary Fig. 11. Mar-type receptors jointly contribute to the robustness of synchronization mechanism. (a-c), Time evolutions of reporter fluorescence for fixed conditions (a, 50µM SA and 0.1 µM IAA), fixed IAA (b, 0.1 µM IAA) or fixed SA (c, 50µM SA). Mean dEGFP trace for all communities is shown in black (left panel), and corresponding heat maps (right panel) for all recorded communities. (**d**, **e**), Response precision (d) (*** p-value = 8.4^{-6}) and phase drift (e) (*** p-values = 7.7^{-6} and 1.9^{-10}) for three different environmental conditions. Note the robust synchrony was present when both chemicals where dynamically changing. A one-way ANOVA with post-hoc Tukey's HSD was used. The description of violin plots is as in Fig. 1f. A heat map legend is as in Fig. 1i.

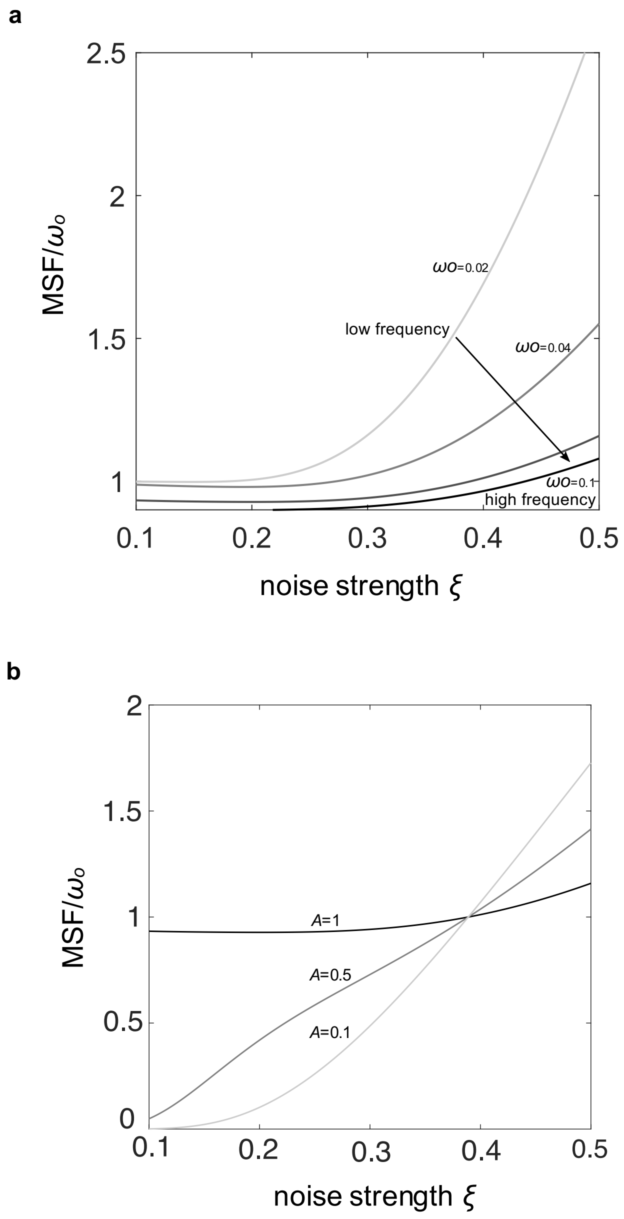

Supplementary Fig. 12. Theoretical model of mean switching frequency (MSF) with rate-dependent hysteresis. (**a, b**) Noisy metastable systems with hysteresis can synchronize MSF to input frequency (ω_0) (MSF/ω is close to 1) for a broad range of noise (ξ). When hysteresis loop grows with ω_0 this system becomes strongly resilient to noise in a broader range (noise gate)(a) as long as the driving amplitude is strong (b).

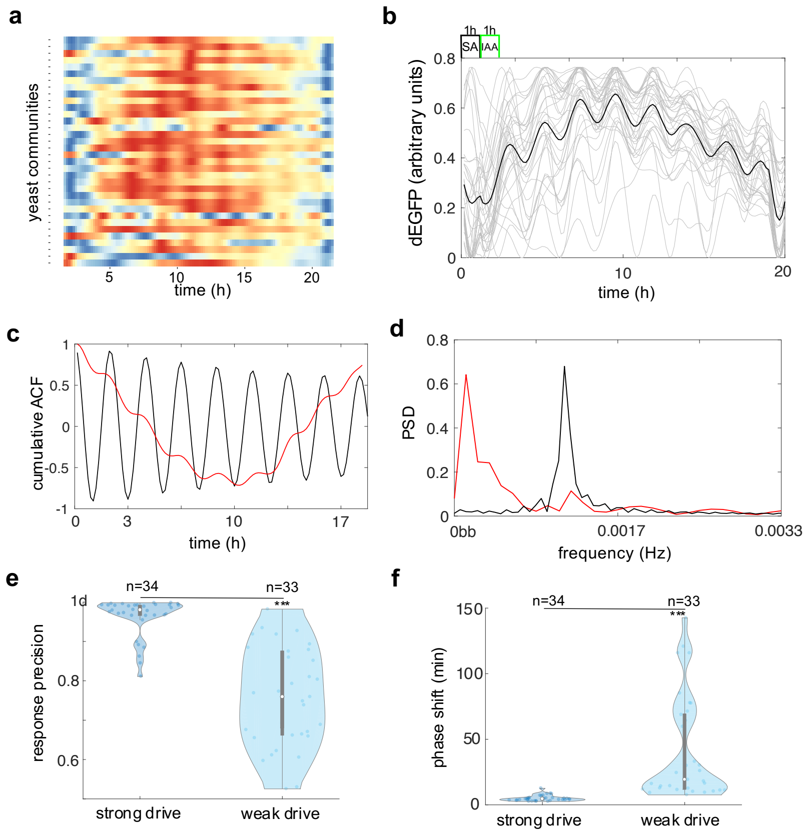

Supplementary Fig. 13. Strong driving is required for robust synchronization. (**a, b**) Heatmap (a) and time evolution of dEGFP (b) for 2-hour cycles between inputs with maximal amplitudes: 50µM SA and 0.1 µM IAA (weak driving). (**c, d**), Cumulative autocorrelation function (mean for all communities) (c) for strong(default) driving (black line, 500 µM and 1 µM), weak driving (red line) of input frequencies. (d) Power spectra analysis reveals frequencies of response that correspond to driving frequencies for strong driving (black line) and weak driving(red line), respectively. (**e**, **f**) Response precision(e) (*** p-value = 1.1^{-10}) and synchronization(f) (phase shift) (*** p-value = 1.2^{-6}) are compromised under weak rhythms. A one-way ANOVA with post-hoc Tukey's HSD was used. The description of violin plots is as in Fig. 1f. A heat map legend is as in Fig. 1i.

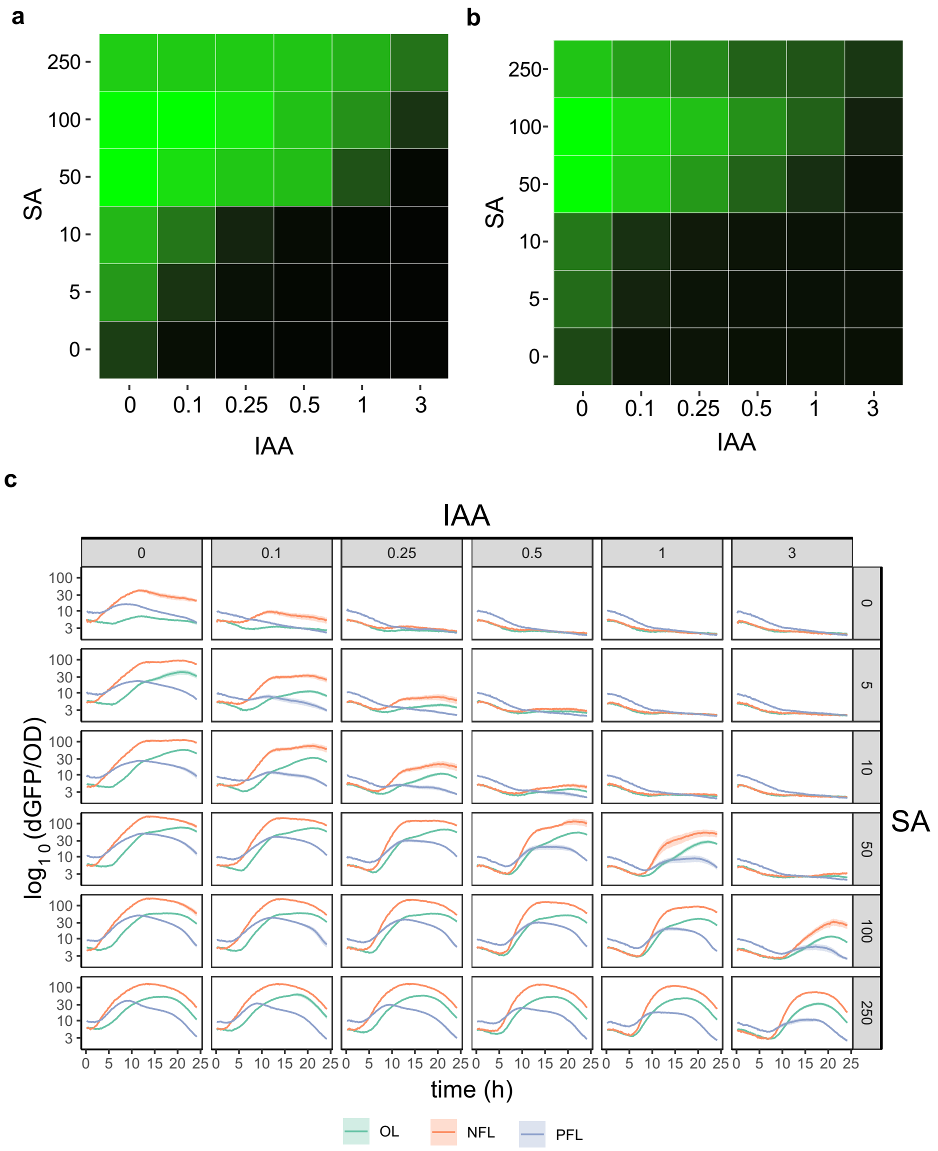

Supplementary Fig. 14. Characterization of NFL and PFL dynamics. (a, b) Heat maps of normalized dGFP fluorescence for different concentration of auxin and SA for Negative feedback loop (NFL)(a) and positive feedback loop (PFL) (b)circuits. (**c**), Time-lapse multiwell plate experiments with graded concentrations of SA and IAA for open loop (OL), NFL and PFL circuits. Note that response time was generally faster in NFL and PFL circuit variants with feedback loops compared to that of the OL circuit. IAA and SA concentrations are in [μM] units.

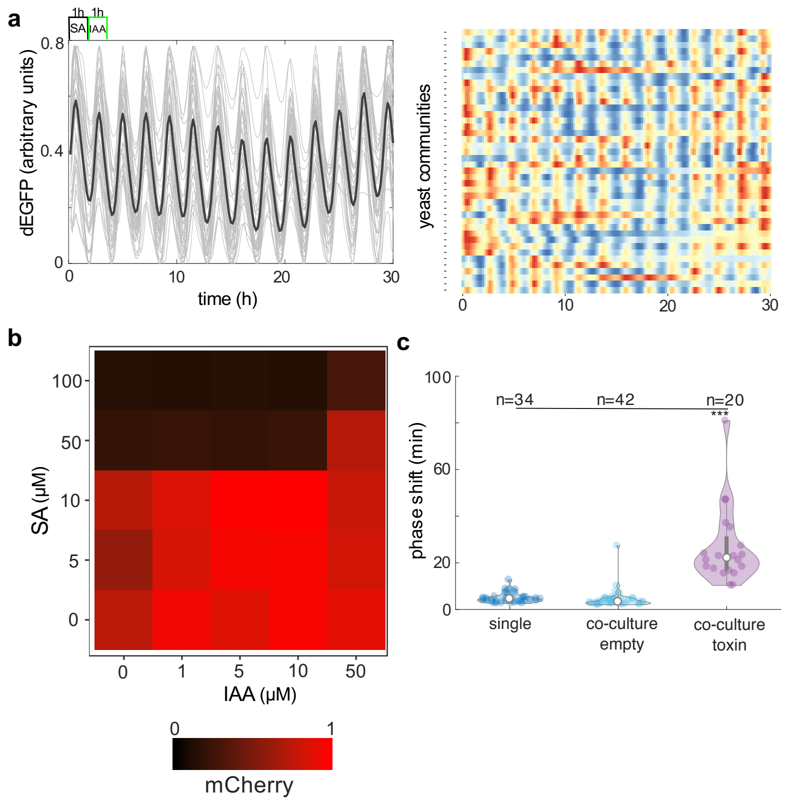

Supplementary Fig. 15. Death portrait and synchronization of sensitive strain in cocultures with and without killer toxin strain. (a), Time evolutions of reporter fluorescence in spatially-distant yeast communities in cocultures with strain carrying empty plasmids. Mean dEGFP trace for all communities is shown in black (left panel), and corresponding heat maps (right panel) for all recorded communities (n=42). **(b)**, Heat map of constitutive mCherry fluorescence of sensitive strain. Note that for high continuously applied amount of SA (above 50μM), large quantities of toxin are released into the ecosystem causing the collapse and death of sensitive strain. (**c**), Phase drift of sensitive strain grown alone, in co-cultured with strain carrying empty plasmids or with killer in co-culture . Note phase drift of less than 25% was observed in coculture experiments, indicating a maintenance of inter-colony synchrony, *** p-value = 6.6^{-10}, a one-way ANOVA with post-hoc Tukey's HSD. The description of violin plots is as in Fig. 1f. A heat map legend is as in Fig. 1i.

Supplementary Table 1. Oligo and synthetic DNA sequences used in the study.

Oligo/synthetic DNA name	sequence
**VECGAD_R**	GCCTTCATGCTCCTTGATTTCC
**GAD_F**	TCTTCATGAAAATATATTACGAGGGCT
**TCYC_F**	CCGCATCATGTAATTAGTTATGTCACG
**HIS_F**	CCGGGCGAATTTCTTATGATTTATGAT
**PMIN_R**	TTAGTGTGTGTATTTGTATTTGCGTGTC
**PMIN_F**	GACACGCAAATACAAATACACACACTAA
**GAL7 promoter**	GGAAATCAAGGAGCATGAAGGCTGTAAAACGACGGCCAGTTCCTTTTGGAAAGCTATA<br>CTTCGGAGCACTGTTGAGCGAAGGCTCATTAGATATATTTTCTGTCATTTTCCTTAACCC<br>AAAAATAAGGGAAAGGGTCCAAAAAGCGCTCGGACAACTGTTGACCGTGATCCGAAG<br>GACTGGCTATACAGTGTTCACAAAATAGCCAAGCTGAAAATAATGTGTAGCTATGTTCA<br>GTTAGTTTGGCTAGCAAAGATATAAAAGCAGGTCGGAAATATTTATGGGCATTATTATG<br>CAGAGCATCAACATGATAAAAAAAAACAGTTGAATATTCCCTCAAAAGACACGCAAAT<br>ACAAATACACACACTAA
**IacR-VP64-ODC**	GACACGCAAATACAAATACACACACTAAATGTTGGACCACTTGGAACAATTCTTGCCA<br>AACAAGGAACCATCTTCTATCCAAAACTTCCCATTCTTCTGGATCTCTCAAGTTAACGG<br>TAAGTACTCTCAATTGATCGAAAAGTCTATCAAGAAGTTGGGTATCGACAACACTAGA<br>AGAAAGATCATCTTGTCTACTAACGCTTTGGGTGAAGCTTCTATCACTGACATCGCTAA<br>CTTGTCTACTTTGAAGTTGACTACTGCTACTAAGGCTGTTTACAGATTGGTTGAAGACG<br>GTATCGTTGAAGTTTACTCTTCTACTACTGACGAAAGAATCTCTATGGTTAAGTTGACT<br>GCTAAGGGTGTTGAATTGGTTGAACAAATCAACCAAATCTCTGTTGTTACTTTGGCTGG<br>TATCTTGAACGCTTTCTCTGAAGACGAATTGCACAACTTGAACCACCAATTGAAGAAGT<br>TGTTCGACTTGATGCCATCTTCTAGCAGGGCTGACGGTTCTGGTAGATCTGGAGTCGAC<br>GGTGGAGGTTCTGACGCATTGGACGATTTTGATCTGGATATGCTGGGAAGTGACGCCCT<br>CGATGATTTTGACCTTGACATGCTTGGTTCGGATGCCCTTGATGACTTTGACCTCGACAT<br>GCTCGGCAGTGACGCCCTTGATGATTTCGACCTGGACATGCTGATTAACGGCAGCCCCA<br>AGAAGAAGAGGAAAGTCGGGAGTCAAAGCCATGGTTTTCCGCCTGAGGTGGAAGAAC<br>AAGATGATGGTACGCTGCCAATGTCATGTGCACAAGAATCCGGGATGGATAGACACCC<br>TGCCGCCTGTGCAAGTGCTCGTATCAACGTGTAATCTTCATGAAAATATATTACGAGGG<br>CT
**MarR-VP64-ODC**	GACACGCAAATACAAATACACACACTAAATGAAGTCTACTTCTGACTTGTTCAACGAA<br>ATCATCCCATTGGGTAGATTGATCCACATGGTTAACCAAAAGAAGGACAGATTGTTGA<br>ACGAATACTTGTCTCCATTGGACATCACTGCTGCTCAATTCAAGGTTTTGTGTTCTATCA<br>GATGTGCTGCTTGTATCACTCCAGTTGAATTGAAGAAGGTTTTGTCTGTTGACTTGGGT<br>GCTTTGACTAGAATGTTGGACAGATTGGTTTGTAAGGGTTGGGTTGAAAGATTGCCAAA<br>CCCAAACGACAAGAGAGGTGTTTTGGTTAAGTTGACTACTGGTGGTGCTGCTATCTGTG<br>AACAATGTCACCAATTGGTTGGTCAAGACTTGCACCAAGAATTGACTAAGAACTTGACT<br>GCTGACGAAGTTGCTACTTTGGAATACTTGTTGAAGAAGGTTTTGCCAAGCAGGGCTGA<br>CGGTTCTGGTAGATCTGGAGTCGACGGTGGAGGTTCTGACGCATTGGACGATTTTGATC<br>TGGATATGCTGGGAAGTGACGCCCTCGATGATTTTGACCTTGACATGCTTGGTTCGGAT<br>GCCCTTGATGACTTTGACCTCGACATGCTCGGCAGTGACGCCCTTGATGATTTCGACCT<br>GGACATGCTGATTAACGGCAGCCCCAAGAAGAAGAGGAAAGTCGGGAGTCAAAGCCA<br>TGGTTTTCCGCCTGAGGTGGAAGAACAAGATGATGGTACGCTGCCAATGTCATGTGCAC<br>AAGAATCCGGGATGGATAGACACCCTGCCGCCTGTGCAAGTGCTCGTATCAACGTGTA<br>ATCTTCATGAAAATATATTACGAGGGCT

**dEGFP(UBG76V-EGFP)**	GACACGCAAATACAAATACACACACTAAATGCAGATCTTCGTTAAAACATTGACCGGG<br>AAAACCATCACTCTAGAGGTTGAACCATCAGACACGATCGAGAATGTTAAAGCTAAAA<br>TTCAAGACAAGGAAGGTATTCCTCCAGACCAACAGCGTTTAATCTTCGCTGGTAAACAA<br>TTGGAAGATGGCAGAACACTTAGCGATTATAACATCCAGAAAGAAAGTACATTGCATT<br>TAGTGCTTCGACTCAGGGGTGTTGTTGGTAAACTCGGAAGACAGGATCCGCCTGTAGCA<br>ACAATGTCTAAAGGTGAAGAATTATTCACTGGTGTTGTCCCAATTTTGGTTGAATTAGA<br>TGGTGATGTTAATGGTCACAAATTTTCTGTCTCCGGTGAAGGTGAAGGTGATGCTACTT<br>ACGGTAAATTGACCTTAAAATTTATTTGTACTACTGGTAAATTGCCAGTTCCATGGCCA<br>ACCTTAGTCACTACTTTCGGTTATGGTGTTCAATGTTTTGCTAGATACCCAGATCATATG<br>AAACAACATGACTTTTTCAAGTCTGCCATGCCAGAAGGTTATGTTCAAGAAAGAACTAT<br>TTTTTTCAAAGATGACGGTAACTACAAGACCAGAGCTGAAGTCAAGTTTGAAGGTGAT<br>ACCTTAGTTAATAGAATCGAATTAAAAGGTATTGATTTTAAAGAAGATGGTAACATTTT<br>AGGTCACAAATTGGAATACAACTATAACTCTCACAATGTTTACATCATGGCTGACAAAC<br>AAAAGAATGGTATCAAAGTTAACTTCAAAATTAGACACAACATTGAAGATGGTTCTGT<br>TCAATTAGCTGACCATTATCAACAAAATACTCCAATTGGTGATGGTCCAGTCTTGTTAC<br>CAGACAACCATTACTTATCCACTCAATCTGCCTTATCCAAAGATCCAAACGAAAAGAG<br>AGACCACATGGTCTTGTTAGAATTTGTTACTGCTGCTGGTATTACCCATGGTATGGATG<br>AATTGTACAAAGGCAGCCCCAAGAAGAAGAGGAAAGTCGGGAGTTAACCGGGCGAAT<br>TTCTTATGATTTATGAT
**synthetic promoter with IacO**	GGAAATCAAGGAGCATGAAGGCAGCTAAGCAATAACTTAGTAAAAAAAGGGTAATTCT<br>ATTATAAACAAAATTTTGCATATTTCCTTGAACGCACATTAGAAAAATTACTTGACAAT<br>TCCATTAAATTTTCTGATATTTTAATTAATGGATAAAAATACATGGAAAAGTGACATTA<br>CTCGCATCCATTCTCAGGCTGGCATATATATATGTGCGCGTATATACATGATTATATGG<br>CATGTATGTGCTCTGTATGTATATAAAACTCTTTTTTTCTTTTTTTCTCTAAATTTTTTTC<br>CTTATACATTAGGACCTTTGCAGCATAAATTACTATACTTCTATAGACACGCAAATACA<br>AATACACACACTAA
**synthetic promoter with MarO**	GGAAATCAAGGAGCATGAAGGCCATTACTCGCATCCATTCTCAGGCTGACTTATACTTG<br>CCTGGGCAATATTATCCCATGCAACTAATTACTTGCCAGGGCAACTAATGTGGCATATA<br>TATATGTGCGCGTATATACATGATTATATGGCATGTATGTGCTCTGTATGTATATAAAA<br>CTCTTTTTTTCTTTTTTTCTCTAAATTTTTTTCCTTATACATTAGGACCTTTGCAGCATA<br>AATTACTATACTTCTATAGACACGCAAATACAAATACACACACTAA
**Synthetic promoter IacO + MarO**	GGAAATCAAGGAGCATGAAGGCAGCTAAGCAATAACTTAGTAAAAAAAGGGTAATTCT<br>ATTATAAACAAAATTTTGCATATTTCCTTGAACGCACATTAGAAAAATTACTTGACAAT<br>TCCATTAAATTTTCTGATATTTTAATTAATGGATAAAAATACATGGAAAAGTGACATTA<br>CTCGCATCCATTCTCAGGCTGACTTATACTTGCCTGGGCAATATTATCCCATGCAACTA<br>ATTACTTGCCAGGGCAACTAATGTGGCATATATATATGTGCGCGTATATACATGATTAT<br>ATGGCATGTATGTGCTCTGTATGTATATAAAACTCTTTTTTTCTTTTTTTCTCTAAATTTT<br>TTTCCTTATACATTAGGACCTTTGCAGCATAAATTACTATACTTCTATAGACACGCAAA<br>TACAAATACACACACTAA
**K1**	GACACGCAAATACAAATACACACACTAAATGACCAAGCCTACGCAGGTTTTGGTCCGT<br>TCAGTCTCCATCTTGTTCTTCATTACTCTATTACATTTGGTAGTTGCACTGAATGACGTA<br>GCGGGTCCCGCAGAAACAGCCCCCGTTTCTCTACTACCTAGAGAAGCCCCCTGGTACGA<br>CAAGATTTGGGAAGTCAAAGACTGGTTGCTACAGAGAGCAACCGACGGAAACTGGGGC<br>AAGTCTATAACGTGGGGTAGTTTCGTGGCAAGTGACGCCGGAGTGGTGATCTTCGGAA<br>TTAACGTGTGCAAAAACTGCGTAGGAGAGAGGAAAGACGACATCAGCACGGATTGTGG<br>AAAACAGACCCTTGCCTTACTTGTGAGTATATTCGTGGCCGTCACGAGCGGTCATCACT<br>TAATTTGGGGAGGTAATAGACCGGTTTCTCAAAGCGACCCCAATGGAGCCACGGTTGC<br>TAGAAGAGACATCAGCACGGTAGCAGATGGGGACATCCCCTTAGACTTTAGTGCTCTA<br>ACATCCCCTTAGACTTTAGTGCTCTAAATGATATACTAAACGAACATGGTATATCCATC<br>TTACCAGCGAATGCGTCACAGTACGTCAAACGTAGTGACACAGCAGAACATACAACCT<br>CTTTCGTAGTAACCAACAACTACACTTCACTACACACTGACCTAATACACCACGGAAAC<br>GGCACCTACACCACGTTCACCACGCCACATATTCCAGCCGTAGCAAAAAGGTATGTTTA<br>CCCGATGTGCGAACACGGCATAAAGGCTAGCTACTGTATGGCGTTGAATGACGCTATG<br>GTTAGCGCTAATGGCAATTTATATGGCCTAGCTGAGAAACTTTTCTCCGAGGATGAGGG<br>CCAATGGGAGACCAATTACTACAAACTTTACTGGTCCACCGGGCAATGGATCATGAGC<br>ATGAAGTTCATCGAAGAGAGCATAGACAATGCAAATAATGATTTCGAAGGGTGCGATA<br>CTGGGCACTAACCGGGCGAATTTCTTATGATTTATGAT
