# Supplementary material for: Synchronization of gene expression across eukaryotic communities through chemical rhythms
Source: Nat Commun. 2021 Jun 29;12:4017. doi: 10.1038/s41467-021-24325-z (PMC8242030; doi:10.1038/s41467-021-24325-z)
Supplement: Supplementary file 7 — Reporting Summary [file 41467_2021_24325_MOESM7_ESM.pdf]

nature research

Corresponding author(s): Krzysztof Wabnik

Last updated by author(s): Jun 13, 2021

Reporting Summary

Nature Research wishes to improve the reproducibility of the work that we publish. This form provides structure for consistency and transparency in reporting. For further information on Nature Research policies, see our Editorial Policies and the Editorial Policy Checklist.

Statistics

For all statistical analyses, confirm that the following items are present in the figure legend, table legend, main text, or Methods section.

n/a	Confirmed	
☐	☒	The exact sample size (n) for each experimental group/condition, given as a discrete number and unit of measurement
☐	☒	A statement on whether measurements were taken from distinct samples or whether the same sample was measured repeatedly
☐	☒	The statistical test(s) used AND whether they are one- or two-sided<br>*Only common tests should be described solely by name; describe more complex techniques in the Methods section.*
☒	☐	A description of all covariates tested
☒	☐	A description of any assumptions or corrections, such as tests of normality and adjustment for multiple comparisons
☐	☒	A full description of the statistical parameters including central tendency (e.g. means) or other basic estimates (e.g. regression coefficient) AND variation (e.g. standard deviation) or associated estimates of uncertainty (e.g. confidence intervals)
☐	☒	For null hypothesis testing, the test statistic (e.g. F, t, r) with confidence intervals, effect sizes, degrees of freedom and P value noted<br>*Give P values as exact values whenever suitable.*
☒	☐	For Bayesian analysis, information on the choice of priors and Markov chain Monte Carlo settings
☒	☐	For hierarchical and complex designs, identification of the appropriate level for tests and full reporting of outcomes
☒	☐	Estimates of effect sizes (e.g. Cohen's d, Pearson's r), indicating how they were calculated

Our web collection on statistics for biologists contains articles on many of the points above.

Software and code

Policy information about availability of computer code

Data collection	Open-source Micro-manager software v.2.0 (https://micro-manager.org/) was used to control imaging conditions and hydrostatic pressure-driven flow system.
Data analysis	Image processing and analysis were performed using Fiji v2.0 , Matlab R2018a and R-studio v4.0.3. Subsequent analysis was performed with custom scripts are available at following link: https://github.com/PlantDynamicsLab/-Synchronization.git. Macromolecular dynamics was performed in OpenMM platform v7.4 and processing and visualization was done in VMD v1.9.3 and UCSF Chimera v1.3. Molecular docking was done with Autodock Vina and structural alignment was performed with Molecular Evolutionary Genetics Analysis (MEGA) 11.

For manuscripts utilizing custom algorithms or software that are central to the research but not yet described in published literature, software must be made available to editors and reviewers. We strongly encourage code deposition in a community repository (e.g. GitHub). See the Nature Research guidelines for submitting code & software for further information.

Data

Policy information about availability of data

All manuscripts must include a data availability statement. This statement should provide the following information, where applicable:

- Accession codes, unique identifiers, or web links for publicly available datasets
- A list of figures that have associated raw data
- A description of any restrictions on data availability

Authors can confirm that all relevant data are included in the manuscript and/or its Supplementary Information files. Raw Data for Fig. 1f, Fig. 3h,i ,Fig. 4d-f, Fig. 5g,h, Supplementary Fig. 2c,d, Supplementary Fig. 4b, Supplementary Fig. 8c, Supplementary Fig. 9d,e, Supplementary Fig. 10d,e, Supplementary Fig. 11d,e, Supplementary Fig. 13e, f, Supplementary Fig. 15c are provided as Source Data. Primer and plasmid part sequences are provided as Supplementary Table 1. Strains and plasmids are available from the corresponding author upon reasonable request.

Field-specific reporting

Please select the one below that is the best fit for your research. If you are not sure, read the appropriate sections before making your selection.

☒ Life sciences ☐ Behavioural & social sciences ☐ Ecological, evolutionary & environmental sciences

For a reference copy of the document with all sections, see nature.com/documents/nr-reporting-summary-flat.pdf

Life sciences study design

All studies must disclose on these points even when the disclosure is negative.

Sample size	Sample size was determined based on experiments to guarantee a statistical significance. For micro-plate reader fluorescence assays 3 replicates per population of cells were performed in accordance with standards for this type of experiments. For microfluidics experiments typically n > 10 traps were independently analyzed per condition as a standard in the field. Experiments were repeated with similar results. The One-way ANOVA with post-hoc Tukey's HSD test was used. Violin plots were used instead of classical box plots. These plots are showing following features: white dot is a median, gray thick bar represents interquartile range and gray line crossing median white dot denotes 95% confidence intervals. Gray contours represents density plot. All data points are shown.
Data exclusions	For time-lapse live imaging, only tissues/cells that died, or did not grow or drifted from imaging area were excluded from data analysis.
Replication	All experiments were repeated at least three times with similar results. Each trap in the microfluidic device represents an independent biological replicate.
Randomization	Randomization was assured at each independent experiments due to random filling of cells in microfludics chambers before each control expriment
Blinding	Researchers were not blinded during experiment execution since experiments were not based on qualitative scoring metrics. All reported data are fluorescent marker measures so blinding does not apply to this study

Reporting for specific materials, systems and methods

We require information from authors about some types of materials, experimental systems and methods used in many studies. Here, indicate whether each material, system or method listed is relevant to your study. If you are not sure if a list item applies to your research, read the appropriate section before selecting a response.

Materials & experimental systems

n/a	Involved in the study
☒	☐ Antibodies
☒	☐ Eukaryotic cell lines
☒	☐ Palaeontology and archaeology
☒	☐ Animals and other organisms
☒	☐ Human research participants
☒	☐ Clinical data
☒	☐ Dual use research of concern

Methods

n/a	Involved in the study
☒	☐ ChIP-seq
☒	☐ Flow cytometry
☒	☐ MRI-based neuroimaging
